# Activities of Stress Axes in Female and Male Patients with Urge Incontinence

**DOI:** 10.3390/jcm15218428

**Published:** 2026-10-29

**Authors:** Fethi Ahmet Uğur, Ümit Uysal, Faruk Dişli, Sedat Yıldız

**Affiliations:** 1 Health Services Vocational School, Health and Care Services Department, Mardin Artuklu University, 47200 Mardin, Turkey; 2 Department of Urology, Health Sciences University Adana City Training and Research Hospital, 01230 Adana, Turkey; uysldr.74@gmail.com; 3 Faculty of Physıcal Therapy and Rehabılıtatıon, Bingöl University, 12000 Bingöl, Turkey; 4 Faculty of Medicine, Basic Medical Sciences, İnönü University, Physiology, 44000 Malatya, Turkey

**Keywords:** urge incontinence, cortisol, heart rate variability (HRV), autonomic nervous system (ANS), hypothalamic–pituitary–adrenal (HPA) axis

## Abstract

**Background/Objectives:** Urge incontinence (UI) is a condition defined as the involuntary loss of urine, affecting both women and men. The underlying pathophysiology is not completely understood, but it might involve the functioning of the autonomic nervous system (ANS) and the hypothalamic–pituitary–adrenal (HPA) axis, i.e., two main stress axes of the body. Therefore, the present study aimed to investigate the association of ANS and HPA axis activities with urge incontinence in female and male genders. **Methods:** For this purpose, four groups were established: (i) female patients with UI (*n* = 57), (ii) female control participants (*n* = 56), (iii) male patients with UI (*n* = 57), and (iv) male control participants (*n* = 57). ANS activity was assessed by heart rate variability (HRV) derived from a 5-min electrocardiogram (ECG) recording, and HPA activity was evaluated by salivary cortisol levels. HRV parameters consisted of both time- and frequency-domain parameters, notably the low frequency/high frequency (LF/HF) ratio, which is commonly interpreted as an index of sympathovagal balance. **Results:** Body mass index and age were higher in UI patients (*p* < 0.001). Cortisol levels did not differ significantly between groups (*p* > 0.05), whereas frequency-domain parameters of HRV (including LF/HF) were significantly lower in patient groups (*p* < 0.05). **Conclusions:** The data suggest that incontinence might be associated with altered ANS activity, with no effect on HPA axis dysregulation. Decreased LF/HF might be associated with a decreased sympathetic urine retention mechanism with increasing age in UI patients. Moreover, the data also suggest that cardiac autonomic control (assessed by HRV) might be a sensitive, non-invasive, and cost-effective tool for studying autonomic control of bladder function.

## 1. Introduction

Urge incontinence (UI) is a disease defined as the involuntary loss of urine [1]. The International Continence Society defines it as unconscious urge leakage that negatively affects quality of life and causes hygienic and social problems. The World Health Organization recognizes incontinence as a priority health issue. Incontinence is a disease that affects both women and men. The prevalence of this disease, which increases with aging, is higher in women than in men. One in every three women experiences urge incontinence during her lifetime [2]. It is stated that 77% of elderly women living in nursing homes are incontinence patients [3]. The prevalence of urge incontinence has been reported to be between 21% and 42% in older women, and between 10% and 28% in men [4]. It is reported to be a disease affecting approximately 6–10% of the world’s population [5]. Incontinence affects the physical, social, and psychological status of individuals and can cause serious economic and medical losses [6]. The annual cost of incontinence in the United States is estimated to be more than 78 billion dollars [7].

In normal micturition, the bladder, urethra, urge sphincters (internal and external), and the autonomic nervous system (ANS) function coordinately to ensure urine storage and the realization of voiding at the desired time [8,9]. While the sympathetic nervous system is active during urine storage, the parasympathetic system becomes active during urination [10]. Although the pathophysiology of urge incontinence is not fully known, it is stated that this disease may originate from hormonal, psychological, physiological, and ANS-related changes [9,11].

Stress refers to the physiological, neuroendocrine, emotional, and behavioral responses of the body to internal and external stimuli [12,13]. While acute stress acts as a defensive adaptation for the organism [14], chronic stress can lead to pathological and psychological syndromes [12]. There are two important axes that manage stress in the body: the ANS and the hypothalamic–pituitary–adrenal (HPA) axis [15]. The ANS is divided into two parts: the sympathetic nervous system and the parasympathetic nervous system. The sympathetic nervous system becomes active in cases of danger, excitement, and stress, while the parasympathetic nervous system shows increased activity during digestive activities and rest. The sympathetic and parasympathetic activity of the autonomic nervous system affects the activity of the cardiovascular system [16]. Heart rate variability (HRV), which is an easy and non-invasive method, is used to determine the activity of the autonomic nervous system. The HPA axis, another important axis activated during stress, causes the release of cortisol hormone from the cortical part of the adrenal gland [17]. Cortisol is an important parameter in determining the activity of the HPA axis [18,19]. Although cortisol can be detected through different biological pathways [20,21,22], it can be practically measured non-invasively from saliva [23]. Salivary cortisol measurement is used as an important marker for the assessment of the HPA axis [24].

Given the increasing prevalence with aging and the rise in average life expectancy, it is evident that the importance of incontinence complaints for human health will increase day by day [25]. Incontinence can cause multifaceted negative effects on human life, such as economic losses, feelings of shame, social isolation, loss of work performance, decreased participation in leisure activities, and impairments in sexual events [26]. Although the storage and evacuation of urine is a complicated event coordinated by the ANS [27], there are insufficient studies on the relationship between stress axes and voiding events [28]. In this study, we aimed to examine the effects of both stress axes (ANS and HPA) in incontinence patients in both men and women. It is anticipated that the data obtained as a result of our study will contribute to the understanding of incontinence pathophysiology and the development of strategies regarding the management of this disease.

## 2. Materials and Methods

Ethical approval was obtained prior to this study (2022/49). Data were obtained from individuals over the age of 18 who applied to the urology outpatient clinic of the Mardin Training and Research Hospital where this study was conducted. For this study, four groups were established: (i) 57 female patients with urge incontinence, (ii) 56 female control participants, (iii) 57 male patients with urge incontinence, and (iv) 57 male control participants (n_total_ = 227). All participants were provided with a Voluntary Informed Consent Form containing detailed information about this study, and necessary briefings were made. Before starting the procedure, each participant was interviewed individually to alleviate states of anxiety and concern, ensuring they were relaxed. Additionally, contact information was collected to manage any adverse situations that might require the re-collection of ECG recordings, blood, or urine samples.

**Figure 1:**
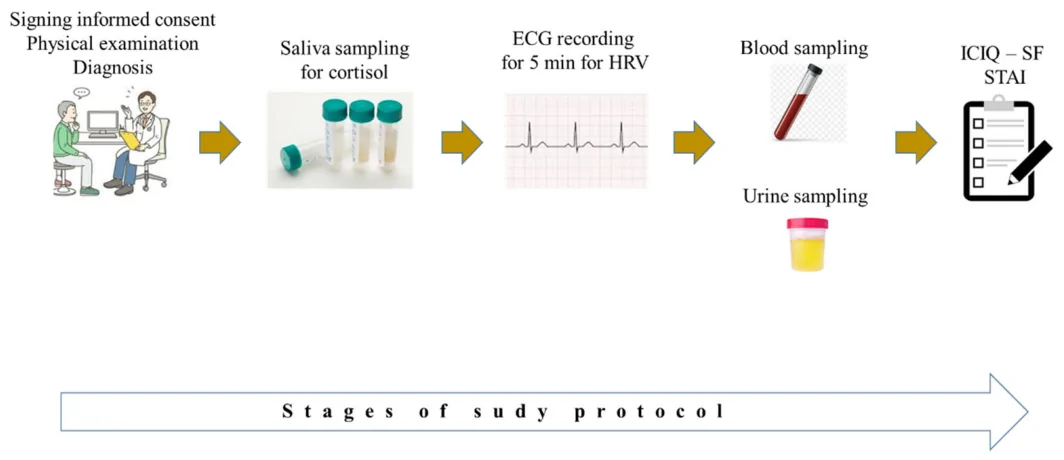
Study protocol. Upon arrival to the hospital, the participants were briefed about this study and were asked to sign the Voluntary Informed Consent Form. They were physically examined by a specialist and provided saliva sample for cortisol analyses. Afterwards, their heart rate variability (HRV) was assessed by 5 min continuous electrocardiogram (ECG). After HRV recordings, participants provided blood and urine samples for determination of hematological parameters (together with blood urea nitrogen and creatinine) and urinalysis. Finally, they filled in the State and Trait Anxiety Inventory (STAI-I and STAI-II) and the International Consultation on Incontinence Questionnaire—Short Form (ICIQ-SF).

The stages of the study protocol are shown in Figure 1. Participants were informed about this study and signed an Informed Consent Form detailing the process. Participants were instructed on saliva collection; they were asked to rinse their mouth thoroughly with water and dry their lips with a paper towel. Saliva was collected into propylene collection tubes (2 mL) using the passive drool method, as described by Ozgocer et al. [29]. In the passive drool technique, participants kept their chin at chest level, allowing saliva to flow passively into the tubes. For participants in need, a mouthpiece made of a propylene cylinder was used to transfer saliva. The tubes were sealed and wrapped with paraffin tape to prevent leakage during transport and evaporation during storage.

Following saliva collection, all participants were asked to sit on a chair and prepare for ECG recordings. For this purpose, limb electrodes were attached, and ECG recordings were taken for 5 min to determine Heart Rate Variability (HRV). Subsequently, they were directed for blood sampling (complete blood count, urea, creatinine) and urine sampling (mucus, leukocytes, erythrocytes, density, pH, bilirubin, ketones, red blood cells, glucose, protein, appearance, color, nitrite). Participants completed the STAI-I (State), STAI-II (Trait), and ICIQ-SF (International Consultation on Incontinence Questionnaire—Short Form) surveys. Blood and urine samples were analyzed in the hospital biochemistry unit immediately after collection. Saliva samples were stored in a refrigerator at −20 °C until analysis.

### 2.1. Questionnaires

Participants completed the State and Trait Anxiety Inventory (STAI-I and STAI-II) and the International Consultation on Incontinence Questionnaire—Short Form (ICIQ-SF). STAI-I and STAI-II are commonly used scales for assessing situational (current) and trait (generalized) anxiety, respectively [30]. The scales include both direct statements (representing negative emotions) and reverse statements (representing positive emotions). Higher scores indicate higher anxiety levels [31]. The ICIQ-SF is a simple, brief, and highly useful questionnaire frequently recommended by the International Continence Society (ICS) for incontinence symptoms [32]. It consists of 6 questions, and the total score is determined by summing the scores of questions 3, 4, and 5. These forms are valuable for determining the degree of symptoms and discomfort before treatment and evaluating the degree of recovery post treatment.

### 2.2. Cortisol Testing and Determination of Cortisol Levels

Salivary cortisol concentrations were determined via a competitive enzyme-linked immunosorbent assay (ELISA) [29].

### 2.3. Blood and Urine Sample Collection

Blood and urine tests were obtained from all participants. For subjects included in the incontinence study, urea, creatinine, and complete blood count parameters were examined. Urea and creatinine levels are used to determine renal function. A complete urinalysis was performed on the collected urine (mucus, leukocytes, erythrocytes, density, pH, bilirubin, ketones, red blood cells, glucose, protein, appearance, color, nitrite). It is noted that performing urine analysis is crucial in patients with urination problems [33]. A complete urinalysis was performed to detect acute urge tract infection, hematuria (which may stem from tumors or infections), proteinuria (which may stem from infections or kidney disease), and glycosuria (which may stem from diabetes).

### 2.4. Measurement of Heart Rate Variability (HRV)

HRV was determined by using the data obtained by a 5 min continuous electrocardiogram (ECG) recording. HRV, measuring cardiac autonomic function, is utilized as a physiological non-invasive marker in the evaluation of autonomic nervous system function [34]. Limb leads were attached to the arms and legs of the participants and HRV was calculated from the ECG recording (Poly-Spectrum 8/E, Neurosoft, Ivanovo, Russia) by using special software (Neurosoft, (Version 6.0), Ivanova, Russia). Participants were asked to turn off or silence their mobile phones and remove jewelry (watches, necklaces, bracelets, etc.) from the lead areas. Using a ECG device, 5 min recordings were taken for all participants in the same environment while sitting normally on a stretcher. Before recording, it was ensured that participants were comfortable and any stress was alleviated; recording began after they felt ready. During the recording, participants were instructed not to speak or move, not to close their eyes for long periods, and to breathe normally.

### 2.5. Statistical Analysis of Data

The General Linear Model (GLM) was used to analyze the data by applying 2 (male vs female) × 2 (healthy vs incontinence) factorial analysis. Gender*group interactions were also statistically identified. The Mann–Whitney U Test was used for categorical data. Spearman’s Rho Test was used for correlation analysis. An alpha value of less than 0.05 (*p* < 0.05) was considered statistically significant. The data are presented as the mean ± standard error unless otherwise is stated.

## 3. Results

### 3.1. Physiological Parameters of Participants

The physiological characteristics of male and female participants in the incontinence and control groups are provided in Table 1. Analysis shows that height (*p* = 0.000) and weight (*p* = 0.001) data significantly differed by gender, while age (*p* = 0.085) and BMI (*p* = 0.134) did not. When physiological parameters were examined by group, significant differences were found across all parameters. Regarding interactions, significant differences were found in height (*p* = 0.019) and age (*p* = 0.000) parameters, while no significant differences were observed in weight (*p* = 0.578) and BMI (*p* = 0.185).

### 3.2. Hematological and Urine Analysis Parameters

Hematological parameters showed gender differences, but no significant difference was found between the descriptive urine analysis and complete urine analysis results of male and female participants.

### 3.3. STAI Questionnaire Data

For STAI-I data, in the female participant groups, in general no significant difference was observed between the incontinence patients and healthy controls, except the latter group being more worried about what was going to happen to them (*p* = 0.005). For male participants, a similar trend was observed, except that the incontinence patients regarded themselves as more irritable compared to the control group (*p* = 0.046).

For STAI-II data, in general no significant difference was observed between the incontinence patients and healthy controls, except that the patients regarded themselves as less happy than the control group (*p* = 0.047). On the other hand, the male participants regarded themselves as less self-confident (*p* < 0.05).

ICIQ-SF (International Consultation on Incontinence Questionnaire—Short Form)—Female: Data are presented in Table 2. A significant difference in ICIQ-SF scores was found between groups, with a median of 0 for the control group and 12 for the patient group (*p* = 0.000). For the question “When does urine leak?”, a significant difference was also found (control median: 1, patient median: 2; *p* = 0.000).

ICIQ-SF (International Consultation on Incontinence Questionnaire—Short Form)—Male: Data are presented in Table 3. A significant difference in ICIQ-SF scores was found between groups (control median: 1, patient median: 12; *p* = 0.000). For the question “When does urine leak?”, a significant difference was also found (control median: 1, patient median: 3.5; *p* = 0.015).

### 3.4. Urea, Creatinine, Cortisol, and HRV Parameters of Male and Female Participants

Urea, creatinine, cortisol, and HRV values are presented in Table 4. Males had higher urea, creatinine and %LF but no other difference was found between the genders. On the other hand, incontinence patients had higher creatinine, urea, RMSSD but lower HR, LF, VLF and LF/HF values. Cortisol levels were not different between the genders and between the patient groups compared to the control groups (*p* > 0.05). No interactions were observed for the parameters presented in Table 4.

### 3.5. Correlations Between Salivary Cortisol and HRV Parameters in the Female and Male Incontinence Groups

No significant correlation was observed between salivary cortisol and HRV values in the female and male incontinence groups (*p* > 0.05).

## 4. Discussion

The present study, investigating the association between stress response systems and UI in both men and women, suggested that ANS activity, rather than the HPA axis, appears to be associated with UI, independent of gender. Thus, the data suggest that ANS activity, as evaluated through HRV, could possess greater clinical relevance in the context of urge incontinence. As the ANS plays a central role in urine withholding and urination processes, the current study reveals that determination of ANS activity by HRV might be a non-invasive, cheap and easy method to study incontinence in both males and females. As discussed below, the current study might provide practically useful preliminary data regarding the etiology, diagnosis, prognosis, and treatment of UI.

### 4.1. UI Questionnaires Successfully Reflected Incontinence in Both Male and Female Patients

The obtained data showed that the ICIQ-SF form successfully identified male and female patients with incontinence. This form appears to be suitable for screening studies to identify patients with incontinence.

### 4.2. Urea and Creatinine Levels Were Higher in UI Patients

When the data were examined, urea and creatinine levels were found to be higher in both male and female patients with incontinence. This situation may be due to the underlying cause of incontinence. In some types of incontinence (like neurogenic bladder), urine is accumulated in the urge bladder due to inadequate emptying of the bladder. In this case, although some dripping from the bladder occurs, there is also a pressure towards the kidneys causing decreased filtration. As the age of the incontinence patients is higher, it is likely that neurogenic control of the bladder might have been compromised. In fact, it might be speculated that decreased sympathovagal balance in these patients (as revealed by LF/HF) might prevent complete or effective emptying of the bladder, and retrogradely increase pressure on the filtration apparatus in the kidneys, culminating in increased blood urea and creatinine levels.

### 4.3. ANS, but Not HPA Axis, Activity Was Associated with Incontinence

ANS function is tightly associated with bladder function and urination. Its sympathetic branch dilates the bladder muscles but constricts the sphincter, thereby allowing urge retention. On the other hand, its parasympathetic branch does the opposite and allows urination. In the current study, the data obtained by cardiac ANS activity, i.e., HRV, reveal that autonomic function of the incontinence patients is shifted towards more parasympathetic activity, suggesting that sphincter function is reduced but bladder muscle contraction activity is increased, favoring urination (or dripping of urine in these patients). This is in accordance with the decreased LF/HF parameter of HRV in the current study. LF/HF has generally been associated with sympathovagal balance [35], and decreased LF/HF suggests that the balance has shifted towards vagal (parasympathetic) activity.

In UI patients, VLF was lower in comparison to their control groups, irrespective of gender. According to Usui and Nishida [36], low VLF has been associated with increased chronic inflammation and has been associated with unfavorable prognosis in many studies. Thus, in the current study, decreased VLF might suggest a bad health outcome and therefore suggests that the underlying cause of incontinence must be eliminated.

The changes observed for the HRV parameters in UI patients, especially LF/HF, suggest that a non-invasive, cheap and easily applicable HRV parameter could be an integral part of the studies associated with urination. In fact, this finding is in accordance with our previous studies which investigated urination postponement resulting in urge bladder filling [37] and urge bladder emptying following urination postponement [38]. Taken together, the current study and the above-mentioned studies suggest that cardiac autonomic control as assessed by HRV might be used to gather information to understand urination problems, which is an underexplored yet very important area for increasing well-being of elderly people. Moreover, it seems that HRV might help in exploring the etiology, diagnosis, prognosis, and treatment of UI and other urination problems in a practical way.

In the current study, HPA axis activity was not different between UI patients and their respective controls. Moreover, there were no gender differences or interactions. This suggest that HPA axis activity is not directly associated with UI. Rather, ANS activity appears to be much more correlated with UI. Possible reasons for this might be due to feedback control of the HPA axis [39]. To the best of our knowledge, this is the first study investigating the activities of both axes in the same patients, and shows that ANS, but not HPA, is closely associated with UI.

### 4.4. Strengths and Limitations of This Study

The large sample size (*n* = 227) constitutes the strength of our study, in addition to the fact that both stress axes (HPA and ANS) were examined together for the first time in both male and female patients with incontinence.

On the other hand, the current study has several limitations. First, the mean age and BMI of the control groups were lower than those of the patient groups. The increase in the prevalence of incontinence in older groups led to a higher mean age and BMI in the patient groups. In order to have a comparable control group, we considered it more appropriate to include younger and healthier individuals rather than older individuals, because increasing age would be associated with comorbidities and increased likelihood of medicine uses for these comorbid diseases. Another limitation of this study was associated with the possible existence of comorbid diseases in the incontinence group, including prolapsus of pelvic organs in women and benign prostatic hyperplasia in men. Due to accessibility limitations under the current scope of this investigation, obtaining these specific data points was not possible. Therefore, new studies in this area should also take into account a subgroup analysis for the women having prolapsus and for men having benign prostatic hyperplasia. A potential limitation of this study was that the medical histories of the participants regarding diabetes mellitus (DM) and other neurological and metabolic disorders were not comprehensively evaluated. Consequently, the potential confounding effects of these underlying conditions on the observed outcomes cannot be entirely ruled out. Moreover, as incontinence is a complex disorder, external influences such as interviewing and filling in questionnaires may have impacts on the parameters studied. The experimental protocol was structured such that the self-report scales were administered after the assessment of ANS and HPA axis activity. On the other hand, in clinical settings, participants are typically interviewed by a specialist physician, and the saliva collection protocol is detailed immediately prior to sampling. In the present study, however, conducting these procedures at a separate time point was unfeasible due to significant challenges in patient follow-up and the critical need to maximize patients’ compliance on the day of their clinical visit. Ideally, for future investigations, it might be recommended that these assessments be conducted during the initial, direct clinical contact whenever viable.

## 5. Conclusions

The current study suggests that ANS activity, rather than the HPA axis, is associated with UI, indicating that an impaired sympathovagal balance may play a significant role in the etiology of incontinence. Furthermore, the HRV technique, which indirectly derives sympathovagal balance from heart rate variability, emerges as a promising and non-invasive tool that could potentially assist in monitoring the development, prognosis, and treatment response of voiding disorders. Nonetheless, given the limitations outlined above, the present findings should be interpreted with caution. Future well-designed studies are warranted to isolate the effects of these potential confounding variables and to establish more definitive causal relationships.

## Figures and Tables

**Table 1:** Physiological characteristics of male and female participants in the incontinence and control groups. Values are given as mean ± standard deviation.

	Female		Male		Significance (*p* Value)		
	Control	Patient	Control	Patient	Gender (G)	Patient (P)	Interaction G*P
Height	163.18 ± 5.84	161.93 ± 5.17	174.90 ± 6.43	169.93 ± 7.22	0.000	0.000	0.019
Age	38.59 ± 12.71	51.18 ± 17.62	35.14 ± 9.95	61.20 ± 15.36	0.085	0.000	0.000
Weight	70.41 ± 13.25	73.88 ± 14.87	75.43 ± 11.64	80.96 ± 15.59	0.001	0.016	0.578
BMI	26.50 ± 5.20	28.20 ± 5.64	24.64 ± 3.44	28.08 ± 5.02	0.134	0.000	0.185

BMI, body mass index.

**Table 2:** ICIQ-SF data for female patients and control groups. Values are given as median (min–max). Question 6 scoring: 1 = Least, 5 = Most.

	Female ICIQ-SF		
	Control	Patient	*p*
ICI-Q Score	0 (0–19)	12 (0–21)	0.000
When does urge incontinence occur?	1 (1–4)	2 (1–5)	0.000

**Table 3:** ICIQ-SF data for male patients and control groups. Values are given as median (min–max). Question 6 scoring: 1 = Least, 5 = Most.

	Male ICIQ-SF		
	Control	Patient	*p*
ICIQ-SF Score	0 (0–17)	12 (0–21)	0.000
When does urge incontinence occur?	1 (1–4)	3.5 (1–5)	0.015

**Table 4:** Urea, creatinine, cortisol, and HRV values for participants. Values are given as mean ± standard deviation.

	Female		Male		Significance (*p* Value)		
	Control	Patient	Control	Patient	Gender (G)	Patient (G)	Interaction G*P
Urea	25.09 ± 8.00	32.71 ± 11.96	29.39 ± 6.32	35.72 ± 11.56	0.006	0.000	0.627
Creatinine	0.72 ± 0.11	0.79 ± 0.19	0.97 ± 0.18	0.99 ± 0.19	0.000	0.040	0.377
Cortisol	19.39 ± 15.28	24.12 ± 23.75	19.88 ± 21.58	19.72 ± 26.02	0.506	0.437	0.406
HR	87.70 ± 11.38	86.46 ± 11.91	88.11 ± 13.33	81.83 ± 11.91	0.198	0.022	0.123
SDNN	37.29 ± 18.36	33.27 ± 30.05	35.89 ± 17.48	37.91 ± 30.25	0.626	0.763	0.365
RMSSD	27.35 ± 17.58	36.29 ± 54.70	26.57 ± 20.54	44.20 ± 56.17	0.522	0.018	0.436
pNN50	6.74 ± 9.09	7.42 ± 17.12	5.78 ± 10.04	8.38 ± 17.46	0.998	0.383	0.609
CV (%)	5.34 ± 2.54	4.63 ± 4.11	5.06 ± 2.00	4.99 ± 3.77	0.922	0.366	0.454
TP	1522 ± 1445	1123 ± 1774	1579 ± 1763	1247 ± 1519	0.681	0.096	0.877
VLF	716 ± 938	365 ± 415	621 ± 586	421 ± 558	0.822	0.002	0.386
LF	488 ± 477	216 ± 287	583 ± 713	322 ± 435	0.138	0.000	0.935
HF	317 ± 325	542 ± 1436	375 ± 670	504 ± 866	0.937	0.153	0.700
LFnorm	61.89 ± 15.89	51.76 ± 22.95	67.65 ± 15.33	50.58 ± 25.63	0.405	0.000	0.207
HFnorm	38.11 ± 15.89	48.24 ± 22.95	32.35 ± 15.33	49.42 ± 25.63	0.405	0.000	0.207
LF/HF	2.38 ± 2.26	1.70 ± 1.57	3.09 ± 2.63	1.95 ± 2.11	0.104	0.002	0.428
%VLF	49.56 ± 17.70	49.20 ± 22.81	44.29 ± 15.85	44.80 ± 23.32	0.075	0.978	0.873
%LF	30.95 ± 12.59	23.58 ± 13.24	36.88 ± 12.21	24.73 ± 14.65	0.047	0.000	0.178
%HF	19.49 ± 11.29	27.21 ± 23.34	18.83 ± 12.90	30.47 ± 24.38	0.609	0.000	0.442

## Data Availability

The data that support the findings of this study are available on request from the author. The data are not publicly available due to privacy or ethical restrictions.
